# Therapeutic interactions between mesenchymal stem cells for healing medication-related osteonecrosis of the jaw

**DOI:** 10.1186/s13287-016-0367-3

**Published:** 2016-08-17

**Authors:** Yuri Matsuura, Ikiru Atsuta, Yasunori Ayukawa, Takayoshi Yamaza, Ryosuke Kondo, Akira Takahashi, Nobuyuki Ueda, Wakana Oshiro, Yoshihiro Tsukiyama, Kiyoshi Koyano

**Affiliations:** 1 Section of Implant and Rehabilitative Dentistry, Division of Oral Rehabilitation, Faculty of Dental Science Kyushu University, 3-1-1 Maidashi, Higashi-ku, Fukuoka 812-8582 Japan; 2 Department of Molecular Cell and Oral Anatomy, Faculty of Dental Science, Kyushu University, Fukuoka, Japan

**Keywords:** Mesenchymal stem cell, Medication-related osteonecrosis of the jaw, Therapeutic mechanism

## Abstract

**Background:**

Mesenchymal stem cells (MSCs) have been isolated from a variety of tissues, including bone marrow, adipose, and mucosa. MSCs have the capacity for self-renewal and differentiation. Reports have been published on the systemic administration of MSCs leading to functional improvements by engraftment and differentiation, thus providing a new strategy to regenerate damaged tissues. Recently, it has become clear that MSCs possess immunomodulatory properties and can therefore be used to treat diseases. However, the therapeutic effect mechanisms of MSCs are yet to be determined. Here, we investigated these mechanisms using a medication-related osteonecrosis of the jaw (MRONJ)-like mouse model.

**Methods:**

To generate MRONJ-like characteristics, mice received intravenous zoledronate and dexamethasone two times a week. At 1 week after intravenous injection, maxillary first molars were extracted, and at 1 week after tooth extraction, MSCs were isolated from the bone marrow of the mice femurs and tibias. To compare “diseased MSCs” from MRONJ-like mice (d-MSCs) with “control MSCs*”* from untreated mice (c-MSCs), the isolated MSCs were analyzed by differentiation and colony-forming unit-fibroblast (CFU-F) assays and systemic transplantation of either d-MSCs or c-MSCs into MRONJ-like mice. Furthermore, we observed the exchange of cell contents among d-MSCs and c-MSCs during coculture with all combinations of each MSC type.

**Results:**

d-MSCs were inferior to c-MSCs in differentiation and CFU-F assays. Moreover, the d-MSC-treated group did not show earlier healing in MRONJ-like mice. In cocultures with any combination, MSC pairs formed cell–cell contacts and exchanged cell contents. Interestingly, the exchange among c-MSCs and d-MSCs was more frequently observed than other pairs, and d-MSCs were distinguishable from c-MSCs.

**Conclusions:**

The interaction of c-MSCs and d-MSCs, including exchange of cell contents, contributes to the treatment potential of d-MSCs. This cellular behavior might be one therapeutic mechanism used by MSCs for MRONJ.

## Background

Medication-related osteonecrosis of the jaw (MRONJ) is defined as exposed necrotic bone in the oral cavity that displays intractable symptoms which cannot be cured for more than 8 weeks, and has never received radiation treatment [1]. This condition appears to be nearly synonymous with bisphosphonate-related osteonecrosis of the jaw (BRONJ) [2]. Nitrogen-containing bisphosphonates (BPs) are widely used anti-bone resorptive medicines, but they are well known to be associated with osteonecrosis of the jaw (ONJ) [3]. The incidence of MRONJ in cancer patients who have received high doses of intravenous BPs, such as zoledronic acid, to inhibit cancer invasion and migration is much higher than that in patients receiving oral BP treatment for osteoporosis. To date, even though several risk factors, including invasive dental procedure, infection, mechanical trauma to the jaw bone, and use of both immunosuppressive and chemotherapy drugs, have connections with the onset of MRONJ [4–6], the mechanisms remain largely unknown. Additionally, because the etiology of MRONJ is not clear the fundamental method of clinical treatment for this disease is not recognized, and therefore novel treatments are required for immediate worldwide application.

Current symptomatic treatment of MRONJ involves conservative clinical approaches, including antibiotics, oral rinses, pain control, and limited debridement with the aim of reducing the stage of necrosis. We reported previously the positive effects of mesenchymal stem cells (MSCs) for the treatment of MRONJ [7, 8]. MSCs can self-renew and have the potential to undergo multidirectional differentiation. MSCs can differentiate into various lineages to secrete multiple cytokines and growth factors and restore their surrounding microenvironment. Therefore, MSCs have great potential for clinical therapy and have many applications in various fields of regenerative medicine [9]. Of significant interest, MSCs are now well documented to be immune-privileged [10] and secretory cells of immunomodulatory factors [11, 12]. MSCs clearly have an immune-regulatory capacity [13], displaying immunosuppressive effects on certain conditions. Therefore, MSCs have the potential to cure inflammatory diseases. To date, the clinical application of MSCs has focused mainly on their potential for regenerative therapy, predominantly for myocardium, bone marrow, skin, bone, and cartilage tissues. More recently, however, immunomodulatory therapy has been trialed and found to be successful for graft-versus-host disease (GVHD), Crohn’s disease, aplastic anemia, cirrhosis, and multiple sclerosis, following systemic administration of MSCs. Indeed, MSCs have a great potential for clinical prevention and treatment of various autoimmune and inflammatory diseases. However, the detailed mechanisms of MSC therapy for any disease remain unknown, despite the large amount of obtained clinical and experimental data.

Essentially, the therapeutic target in MSC therapy has not been identified. Previous papers have reported MSC abnormalities from ovarectomy (OVX), scleroderma, and systemic lupus erythematosus (SLE) mouse models [14–16]. MSC abnormalities include partial changes in stemness, differentiation potential, proliferation, and colony formation. In some cases, therefore, the application of MSCs may worsen a patient’s condition. Namely, “disease treatment” may in fact mean “diseased MSC treatment”, and therefore it seems comprehensive that “diseased MSCs” (d-MSCs) are made the targets of treatment.

The relationship between MSC-based therapy and MSC function is not well understood. In this study, we hypothesized that administered MSCs may influence the host-diseased MSCs as the therapeutic target to act more efficiently to treat the disease. To test this hypothesis, we analyzed the therapeutic effect of MSCs with various inflammatory properties using a MRONJ mouse model to identify the mechanism of MSC therapy. This study aimed to examine the effectiveness and mechanism of MSC therapy for MRONJ, as well as investigating the pathogenesis of this disease.

## Methods

### Animals

C57BL/6 N mice (male, 6 weeks old; Kyudo Lab, Tosu, Japan) (*n* = 49) and GFP-transgenic C57BL/6 N mice (CAG-EGFP) (male, 6 weeks old; Japan SLC, Shizuoka, Japan) (*n* = 8) were used in this study. All animal experiments were performed under an institutionally approved protocol for the use of animal research at Kyushu University (approval number: A25-124-0).

### Generation of diseased mouse model

**Fig. 1:**
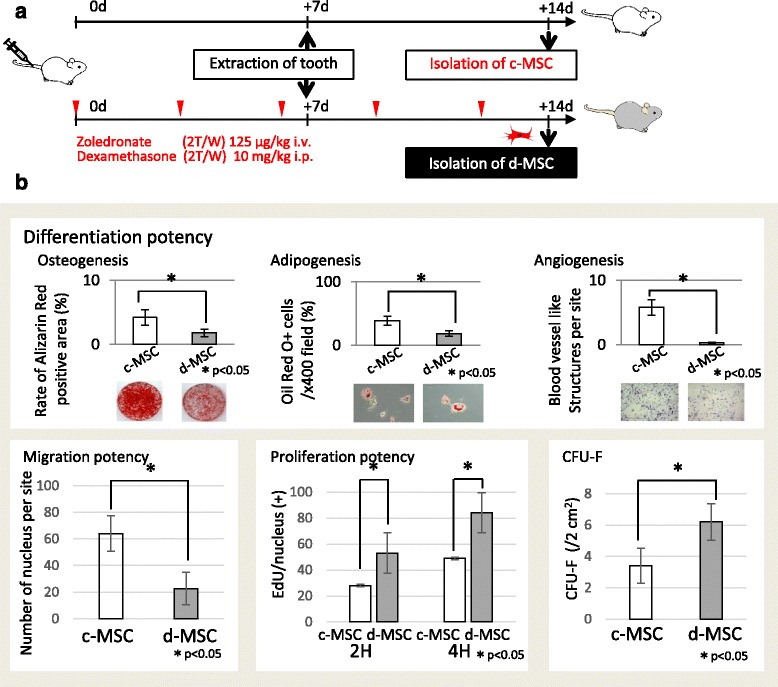
Multipotential differentiation of MSCs. **a** Experimental protocol for the in-vivo study. To isolate diseased MSCs (*d-MSCs*), wild-type C57BL/6 N mice received both Dex and Zol administered intravenously for 2 weeks with tooth extraction. Control MSCs (*c-MSCs*) were isolated from normal mice, which received no treatment (*n* = 4 × 2 groups). **b** (*Top left*) Osteogenic differentiation of MSCs. After culture under osteogenic differentiation conditions for 4 weeks, osteogenic differentiation was determined by Alizarin Red S staining. Quantification of the Alizarin Red S dye content in differentiated osteoblasts from independent experiments is shown (mean ± SD). Scale bar, 50 μm. (*Top middle*) Adipogenic differentiation of MSCs. After culture under adipogenic differentiation conditions for 2 weeks, adipocyte differentiation was determined by Oil Red O staining. Zol treatment decreased adipogenic differentiation of MSCs, as indicated by the decreased number of Oil Red O-positive cells (mean ± SD). *P < 0.05. (*Top right*) Endothelial differentiation of MSCs. After culture under endothelial differentiation conditions for 2 weeks, differentiation was determined by the formation of typical vessel-like-structures. Scale bar, 50 μm. (*Bottom left*) MSC migration determined by fluorescence microscopy. Larger numbers of c-MSCs went through the transwell insert compared with d-MSCs. (*Bottom middle*) Rate of proliferation as determined by the EdU assay. The number of EdU-positive cells was significantly decreased in the d-MSCs from the Zol-treated group compared with c-MSCs from the normal group. (*Bottom right*) MSCs from normal mice generated fewer CFU-Fs compared with MSCs from MRONJ-like mice. *CFU-F* colony-forming unit-fibroblast, *d* days, *EdU* 5-ethynyl-2′-deoxyuridine, *i.p.* intraperitoneal, *i.v.* intravenous

Mice received intravenous (i.v.) zoledronate (Zometa (Zol), 125 μg/kg; Novartis Oncology, East Hanover, NJ, USA) and dexamethasone (Dex, 10 mg/kg; Sigma, St. Louis, MO, USA) twice a week via the tail vein. One week after i.v. injection, maxillary first molars were extracted under systemic chloral hydrate and local lidocaine hydrochloride (Abbott Laboratory, North Chicago, IL, USA) anesthesia. One week after tooth extraction, mice were sacrificed under systemic chloral anesthesia. A total of five doses of Zol and Dex were administered. Untreated mice with tooth extraction were used as a control (*n* = 5) (Fig. 1a).

The intact maxilla, kidney, liver, lung, spleen, femur, and tibia were harvested en bloc. In parallel, peripheral blood was collected for cytokine analysis.

### ELISA of inflammation factors in blood

Peripheral blood was collected from the retro-orbital plexus of mice and centrifuged to obtain the blood serum [17]. Culture supernatants from MSCs were collected [8]. MSCs were extracted using M-PER® mammalian protein extraction reagent. The samples were centrifuged and used in an enzyme-linked immunosorbent assay (ELISA) for detection of interleukin (IL)-2, IL-6 and IL-10 (R&D Systems, Minneapolis, MN, USA).

### Histomorphometry

After each experimental period, each organ was removed from euthanized mice and immersed in 4 % paraformaldehyde (PFA; pH 7.4) for 2 days. The maxilla and femur were then decalcified in 10 % tetrasodium ethylenediaminetetraacetate (EDTA). All samples were dehydrated in increasing concentrations of ethanol, embedded in paraffin, and sectioned in the coronal plane. The sections were stained with hematoxylin and eosin (H & E).

### Isolation and culture of MSCs

MSCs were isolated from the bone marrow of mice as described previously [18]. Briefly, bone marrow cells were flushed out of the mice femur and tibia bone cavities. The cells were passed through a 40-μm cell strainer to obtain a single cell suspension. The single cells were seeded at 1 × 10^6^ cells/dish in 100-mm culture dishes. At 1 day after seeding, the cells were washed with phosphate-buffered saline (PBS) and cultured in growth medium consisting of alpha-minimum essential medium (α-MEM; Invitrogen, Grand Island, NY, USA) containing 20 % fetal bovine serum (FBS; Equitech-Bio, Kerrville, TX, USA), 2 mM l-glutamine (Invitrogen), 100 U/ml penicillin, and 100 μg/ml streptomycin (Invitrogen). After 1 week of culture, the colony-forming unit-fibroblasts (CFU-Fs) had formed colonies. The adherent mesenchymal cells in these colonies were detached by trypsin/EDTA (Invitrogen), reseeded as new cultures, and expanded for further studies.

### Osteogenic differentiation assay

MSCs (passage 2, 5 × 10^5^ cells/dish) were grown on 35-mm dishes to confluence in growth medium and then cultured in osteogenic culture medium (growth medium containing 1.8 mM KH_2_PO_4_ and 10 nM Dex; both Sigma-Aldrich). After 28 days of osteogenic induction, the cultures were stained with a 1 % Alizarin Red S solution (Sigma-Aldrich).

### Adipogenic differentiation assay

MSCs (passage 2, 5 × 10^5^ cells/dish) were grown on 35-mm dishes to confluence in growth medium and then cultured in adipogenic culture medium (growth medium containing 0.5 mM isobutylmethylxanthine, 60 μM indomethacin, 0.5 μM hydrocortisone, and 10 μg/ml insulin; all Sigma-Aldrich). After 14 days of adipogenic induction, the cultures were stained with Oil Red O. The Oil Red O-positive lipid droplets were observed using an inverted microscope (BZ-9000; Keyence, Osaka, Japan).

### Angiogenesis assay

MSCs (passage 2, 5 × 10^5^ cells/dish) were grown on 35-mm dishes to confluence in growth medium and then cultured in endothelial culture medium (growth medium containing 0.5 mM isobutylmethylxanthine (Sigma-Aldrich), 2 mM l-glutamine (Invitrogen), 55 μM 2-mercaptoethanol (Invitrogen), 100 U/ml penicillin, and 100 μg/ml streptomycin (Invitrogen)). After 14 days of endothelial induction, the cultures were stained with toluidine blue (Merck, Darmstadt, Germany). The formation of capillary-like structures (CLS) was observed using an inverted microscope.

### Migration assay

MSCs (1 × 10^4^) were seeded onto a FluoroBlok Insert with an 8.0-μm pore size (Corning, Corning, NY, USA) in a 24-well plate (BD Biosciences, Franklin Lakes, NJ, USA). After 2 days of culture, MSCs on the bottom of the FluoroBlok Insert were fixed with 4 % PFA (Merck) for 5 minutes at room temperature, and then stained with TRITC-phalloidin (1:400) for 2 hours. The cells were washed with 1 % PBS and mounted with VECTASHIELD Mounting Medium containing 4′,6-diamidino-2-phenylindole (DAPI; Vector Laboratories, Burlingame, CA, USA).

### Proliferation assay

Cell proliferation was analyzed by a Click-iT® Plus EdU Imaging Kit (Thermo Fisher Scientific, Waltham, MA, USA). Cultured cells were exposed to 5-ethynyl-2′-deoxyuridine (EdU) for 2 or 4 hours and then fixed in 4 % PFA for 15 minutes followed by the inmation of a 0.5 % Triton® X-100. Fixed cells were treated with the Click-iT® reaction cocktail and phalloidin (1:100) for 15 minutes and then mounted with DAPI-containing medium.

### CFU-F assay

The CFU-F assay was performed as described previously [19]. Passage 1 MSCs were seeded in culture dishes (Nalge Nunc, Rochester, NY, USA). After 16 days of culture, the cells were stained with a solution of 0.1 % toluidine blue and 2 % PFA. Total colony numbers were counted per dish. Three independent experiments were performed.

### MSC injection

MSCs isolated from bone marrow were cultured and passaged three times. These cells were injected (1 × 10^6^ cells) into the mice with or without Dex/Zol via the tail vein (*n* = 5 in each group) at 24 hours after tooth extraction. In the control group, the mice received PBS (*n* = 5).

### Cell cultures

For in-vitro assessment, MSCs (2 × 10^4^/dish) were seeded into 35-mm dishes and incubated with or without the other type of MSCs for 12 hours at 37 °C with 5 % CO_2_. Some MSCs were incubated in medium containing 100 nM chloromethyltetramethylrodamine (Mito, Mitotracker Orange CMTMRos; Molecular Probes, Eugene, OR, USA) for 20 minutes at 37 °C in the dark. After three washes with medium, the cells were examined under an inverted fluorescence microscope.

### Tissue preparation

For in-vivo assessment, tissues were prepared according to the methods described in our previous reports [20, 21]. At the end of each experimental period, mice were deeply anesthetized and perfused intracardially with heparinized PBS, followed by 4 % PFA (pH 7.4). The maxillae were demineralized in 5 % EDTA for 4 days at 4 °C. The prepared site was cut into 10-μm bucco-palatal sections with a cryostat at −20 °C.

### Immunohistochemistry

Immune-fluorescent staining of both in-vitro and in-vivo samples was carried out as follows. Samples were blocked with normal serum matched to secondary antibodies for 1 hour followed by incubation with mouse anti-rat green fluorescent protein (GFP) and CD90 antibodies (1:100; Sigma-Aldrich) overnight at 4 °C. Slides were then treated with FITC-conjugated secondary antibody (1:200; Jackson Immuno Research, West Grove, PA, USA) for 1 hour at room temperature (RT) and mounted with DAPI.

### Statistical analysis

Data are expressed as means ± SD. One-way analysis of variance with Fisher’s least-significant difference test was performed. *P* < 0.05 was considered significant. Experiments were performed using triplicate samples and were repeated three or more times to verify their reproducibility.

## Results

### MRONJ-like mouse model

Figure 1a details the experimental schedules and methods of our study. d-MSCs were isolated from the bone marrow obtained from the femurs and tibias of wild-type C57BL/6 N mice, which received both Dex and Zol administered intravenously for 2 weeks. For the control-MSCs (c-MSCs), the mice did not receive drug treatment before MSC isolation.

### Characteristics of d-MSCs

d-MSCs from MRONJ mice had a higher proliferation rate compared with c-MSCs from normal mice, as estimated by EdU exposure and CFU-Fs (Fig. 1b). In d-MSCs, mineralized nodule formation stained by Alizarin Red S and the number of Oil Red O-positive cells and blood vessel-like structures per site/migrating cells were decreased following treatment with Zol and Dex. These data show that MRONJ disease is caused at the cellular rather than tissue level, for example by MSCs.

### Symptoms of the disease model and therapeutic efficacy of MSCs

**Fig. 2:**
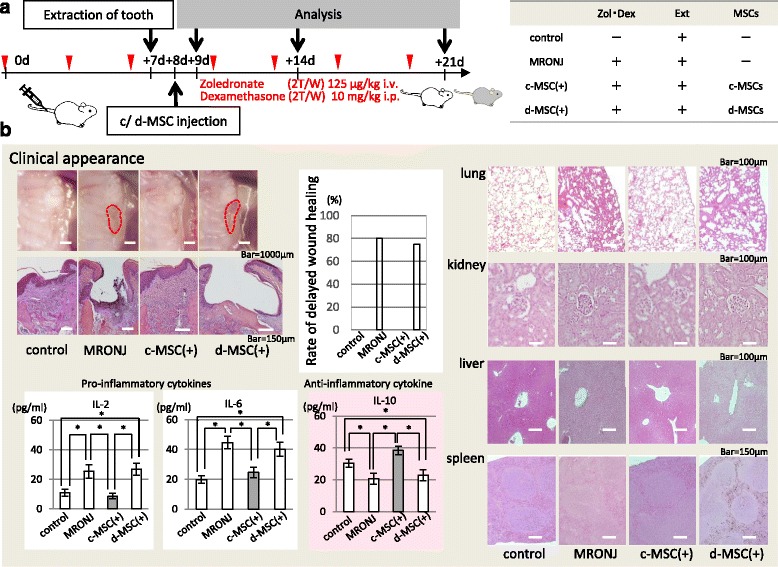
Epithelial healing after tooth extraction in a histological study. **a** Experimental protocol for the in-vivo experiments. Wild-type C57BL/6 N mice received both dexamethasone (*Dex*) and zoledronate (*Zol*) administered intravenously for 2 weeks. c-MSCs or d-MSCs were injected into the mice a day after tooth extraction (*c-MSC(+)* and *d-MSC(+)*). Control mice received no treatment and MRONJ mice received both Dex and Zol but had no MSC treatment. **b** MRONJ model mice without treatment and with diseased MSCs had a lack of epithelial lining in the alveolar socket. In contrast, 100 % of the MSC-treated mice showed complete epithelial coverage, which was confirmed by the histological study. Moreover, MRONJ mice had higher IL-2 and IL-6 levels, and lower IL-10 levels than the controls. Inflammatory cytokine levels of the c-MSC(+) model were similar to those of the controls. The lung, kidney, liver, and spleen of the MRONJ model had obvious destruction of the typical structure, invasion of inflammatory cells, and necrosis of components. These inflammatory organ characteristics were recovered by c-MSCs but not d-MSCs. *n* = 5 × 4 groups. *c-MSC* control MSC, *d* days, *d-MSC* diseased MSC, *i.p.* intraperitoneal, *i.v.* intravenous, *MRONJ* medication-related osteonecrosis of the jaw, *MSC* mesenchymal stem cell

Figure 2b shows development of the MRONJ model and the effects of MSCs on this model. Our experimental data revealed incomplete mucosal healing and open sockets with exposed bone in 80 % of mice treated with a combination of Dex and Zol, but importantly no mice treated with systemic MSCs showed incomplete mucosal healing or open sockets with exposed bone.

Next, we investigated the effects of MSC treatment on the levels of serum inflammatory cytokines, IL-2, IL-6, and IL-10 in MRONJ mice. In this particular case, MRONJ mice had higher IL-2 and IL-6 levels, and lower IL-10 levels than the controls. Moreover, these inflammatory cytokine levels in the c-MSC(+) model were similar to those in the controls. We identified abnormalities in several organs, including the lung, kidney, liver, and spleen, in the MRONJ model. All organs had obvious destruction of the typical structure, invasion of inflammatory cells, and necrosis of components. Interestingly, these inflammatory organ characteristics were recovered by c-MSCs but not d-MSCs.

Development of MRONJ in these treated mice was confirmed by a histological study that revealed a lack of epithelial lining in the alveolar socket. Moreover, necrotic bone was found adjacent to the area of intense inflammatory infiltrates, suggesting an association between inflammation and tissue necrosis in MRONJ-like disease. In contrast, 100 % of the MSC-treated mice showed complete epithelial coverage, which was confirmed by the histological study. In addition, abnormal MSCs from disease model mice were converted to normal MSCs after c-MSC treatment of MRONJ as shown in Fig. 4. These data indicated that MRONJ was caused by abnormal MSCs and could be recovered by treatment with normal MSCs. These data indicated that a cumulative dosage of Zol and Dex may be associated with the occurrence of MRONJ in mice undergoing dental extraction.

### Accumulation of MSCs in the whole body

**Fig. 3:**
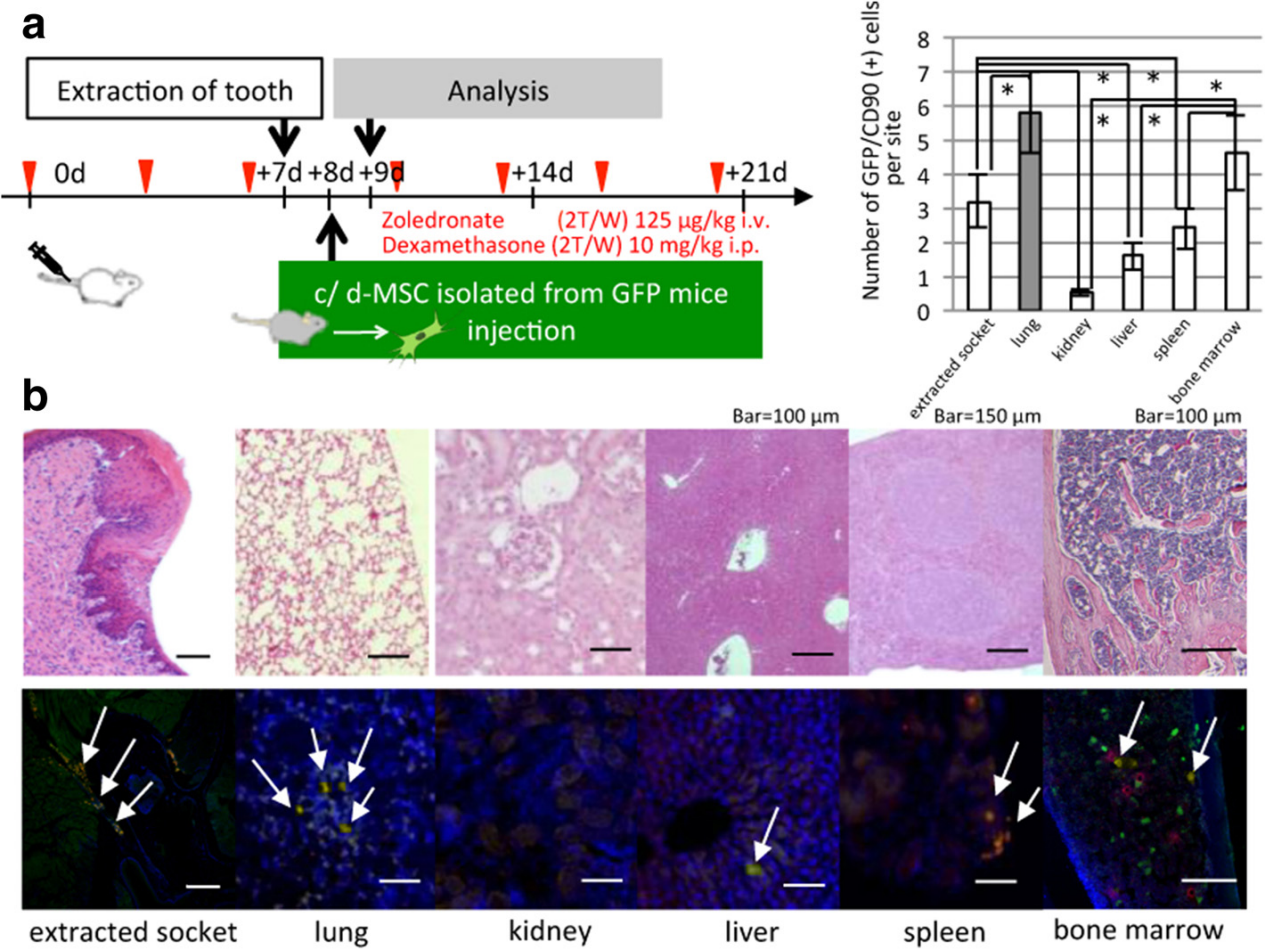
Accumulation of GFP-transgenic injected MSCs into diseased mice. **a** Experimental protocol for the in-vivo experiments. Wild-type C57BL/6 N mice received both Dex and Zol administered intravenously. MSCs from GFP-transgenic mice were injected into the mice 1 day after tooth extraction. **b** In various organs and around the extraction site, injected MSCs (CD-90/GFP-FITC double-positive cells) selectively accumulated into the connective tissue close to the blood vessels. However, no double-positive MSCs were observed in the kidney and gingival mucosa. Scale bar, 100 μm. *n* = 5 × 2 groups. *c-MSC* control mesenchymal stem cell, *d* days, *d-MSC* diseased mesenchymal stem cell, *GFP* green fluorescent protein, *i.p.* intraperitoneal, *i.v.* intravenous. The right panel showed graphially the number of GFP and CD90 positive cells in each organs. Bars represent the mean ± SD of six independent experiments. *P<0.05

As shown in Fig. 3b, we investigated how intravenously transplanted MSCs were able to influence endogenous MSCs in mouse models or in patients. A number of different theories for the mechanism of MSC treatment have been discussed. In a recent study, accumulating MSCs at injured tissue sites in various diseases were found to have direct contact with the injured cells, and controlled the disorganization by promoting the repair and regeneration of the cells and tissue. We therefore decided to conduct the subsequent experiment to examine cell-to-cell contact of MSCs with other cell types.

We showed the ability of i.v. transplanted MSCs to accumulate in various organs, including the dental extraction site. Many CD-90/GFP-FITC double-positive cells had accumulated in the lung, liver, spleen, and extraction site 1 day after MSC injection.

### Intercellular exchange of MSCs

Some previous studies detail the transfection of mitochondria with microRNAs from MSCs to injured MSCs. Based on this consideration, we hypothesized that mitochondria were important for the therapeutic abilities of MSCs to regulate or cure injured cells. The next experiment investigated whether mitochondria have any relationship with MSC therapy. Figure 5 shows in-vitro analysis of d-MSCs and c-MSCs with red-stained mitochondria in direct coculture with GFP-expressing c-MSCs or d-MSCs, respectively. Interestingly, the red-stained mitochondria from c-MSCs were observed to actively move to d-MSCs. This result was consistent with the in-vivo study. As shown in Fig. 6b, many CD90(+) and GFP(−) cells with stained mitochondria were located in the upper portion of the socket. These results indicated mitochondrial exchange between exogenous and endogenous MSCs in MRONJ model mice. These data therefore indicate that MSCs may be able to cure d-MSCs via mitochondrial exchange.

## Discussion

In this study, establishment of the MRONJ mouse model was carried out based on our previous report [7]. Because MRONJ is associated with high doses of BPs, Zol was administered at a dose at least 10 times greater than that used for humans to efficiently induce MRONJ (Fig. 1). Furthermore, the incidence of MRONJ-like disease in mice is enhanced by the combination of BP and Dex compared with Zol alone [7, 22]. Our method therefore ensured development of MRONJ to investigate clinical treatments.

To verify the development of MRONJ in our mouse model, maxillary bones were evaluated for clinical symptoms of MRONJ (Fig. 2), based on guidelines established by the American Association of Oral and Maxillofacial Surgeons and the American Society for Bone and Mineral Research [1]. MRONJ model mice with open sockets and exposed bone were further assessed for MRONJ development using histological and biochemical methods to detect increased inflammation (Fig. 2). These procedures were carried out in a similar manner to a previous study in which inflammatory cells and necrotic bone areas with empty lacunae, fibrosis, and a lack of epithelial lining overlaid areas of dead bone [23].

**Fig. 4:**
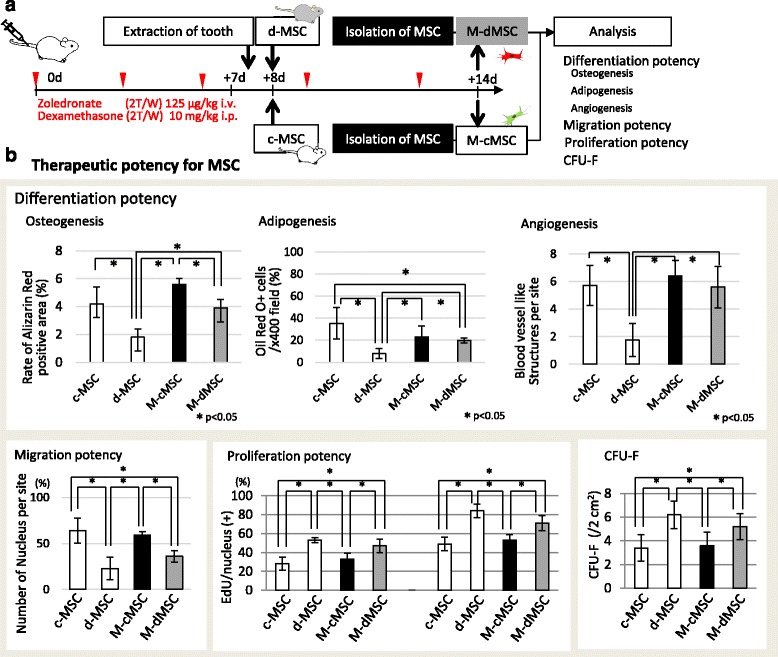
Characteristics of MSCs from MRONJ mice treated with MSCs. **a** Experimental protocol. Wild-type C57BL/6 J mice received both Dex and Zol administered intravenously for 2 weeks (*n* = 5 × 2 groups). MSCs were isolated from MRONJ model mice treated with c-MSCs or d-MSCs a day after tooth extraction (*M-cMSC* and *M-dMSC(+)*). **b** MSC abnormalities in MRONJ were reversed by c-MSCs but not d-MSCs. *CFU-F* colony-forming unit-fibroblast, *c-MSC* control MSC, *d* days, *d-MSC* diseased MSC, *EdU* 5-ethynyl-2′-deoxyuridine, *i.p.* intraperitoneal, *i.v.* intravenous, *MRONJ* medication-related osteonecrosis of the jaw, *MSC* mesenchymal stem cell

Moreover, as shown in Figs. 2b and 4b, d-MSCs from MRONJ mice had abnormalities compared with c-MSCs from normal mice, and the d-MSCs recovered after MSC therapy. These data showed that MRONJ disease occurs at the cellular level rather than the tissue level and can be treated by cellular therapy such as MSC therapy. Although MSCs were originally isolated from bone marrow [9, 24] they are present in many other tissues owing to their perivascular location, including adipose, liver, lung, skeletal muscle, kidney, gingival, and dental pulp tissues [25]. Previous reports show that the effect of systemic MSC administration is affected by the cell source, recipient age, sex, and health status [26, 27]. Moreover, it has been reported that abnormal MSCs are observed in disease models as shown in Figs. 1b, 2b, and 4b. More recently, differences in MSC characteristics have been investigated under each recipient condition. For example, MSCs obtained from females exhibit a higher proliferation rate than those from males [28]. Although MSCs have been shown to exert an inhibitory effect on cancer cells [29], some reports show that MSCs themselves promote the progression of disease symptoms in experimental mice [8, 30, 31], as shown in Fig. 2b. Thus, there appears to be a strong interaction between the recipient condition and the characteristics of their MSCs.

**Fig. 5:**
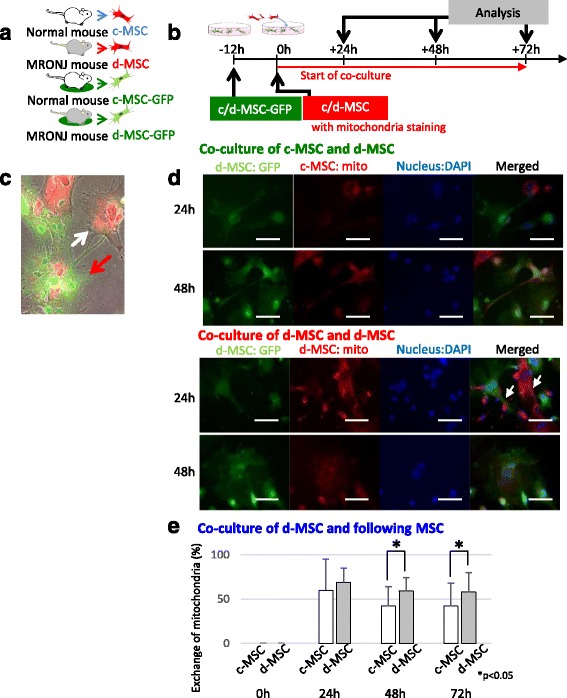
Intercellular exchange of MSCs. **a** Explanatory schema of the various MSCs in this experiment (*n* = 3 × 4 groups). **b** Experimental protocol for the in-vitro experiments. d-MSCs were cocultured with c-MSCs or d-MSCs for 24, 48, and 72 hours to analyze intercellular exchanges between MSCs. **c** Typical image of MSCs with mitochondria exchange. MSC-GFP(+), *green*; MSC-mito(+), *red*. **d**, **e** d-MSCs stained (*red*) for visualization of mitochondria were placed into direct coculture with c-MSCs stained with GFP (*green*). The mitochondria from the c-MSCs moved actively to the d-MSCs. Scale bar, 50 μm. White arrows show the intercellular exchanges in early stage. *c-MSC* control MSC, *DAPI* 4′,6-diamidino-2-phenylindole, *d-MSC* diseased MSC, *GFP* green fluorescent protein, *i.p.* intraperitoneal, *i.v.* intravenous, *MRONJ* medication-related osteonecrosis of the jaw, *MSC* mesenchymal stem cell

**Fig. 6:**
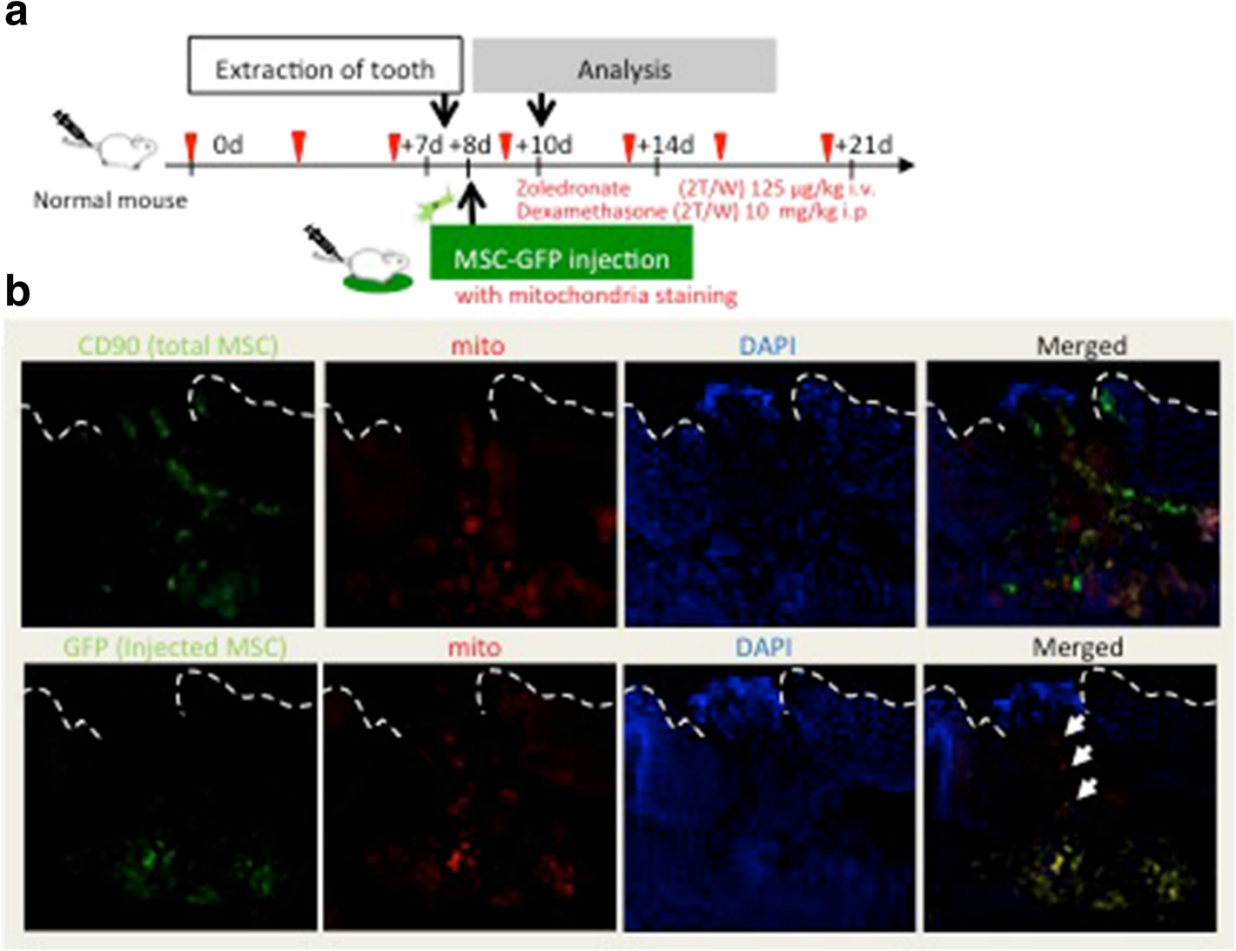
Intercellular exchange between endogenous and exogenous MSCs. **a** Experimental protocol for the in-vivo experiments. Wild-type C57BL/6 N mice received both Dex and Zol administered intravenously. MSCs from GFP-transgenic mice were injected into the mice 1 day after tooth extraction. **b** A pair of continuous sections was considered the same section. A section was stained with an anti-GFP antibody, while the other was stained with an antibody against CD90 and subjected to mitochondrial staining. As a result, many CD90(+) (*total MSCs*) and GFP(−) cells (*endogenous MSCs*) with stained mitochondria were located at the upper portion of the socket (white arrows). Mitochondrial transfer from exogenous MSCs to endogenous MSC was observed in MRONJ model mice. *n* = 5 for sample, *n* = 3 for MSC injection. *d* days, *DAPI* 4′,6-diamidino-2-phenylindole, *GFP* green fluorescent protein, *i.p.* intraperitoneal, *i.v.* intravenous, *MSC* mesenchymal stem cell

Our study showed MSC abnormalities in the MRONJ model, and MSC recovery in the MSC-treated model was confirmed to be similar to that of normal MSCs (Fig. 5). The administered MSCs therefore normalized the d-MSCs in the diseased tissue and improved the diseased environment by exerting therapeutic effects. At present, mechanisms of such therapeutic effects are being investigated. Some studies suggest that administered MSCs affect organs directly, while other studies indicate that administered MSCs secrete growth factors and cytokines that act indirectly through a paracrine mechanism. To more clearly identify the specific mechanism, we focused on mitochondrial exchange among MSCs. A recent study showed that accumulation of MSCs in the damaged tissue sites of various diseases results in direct contact with the injured cells, control of disorganization, and promotion of repair and regeneration [32]. Furthermore, transfer of mitochondria with microRNAs from MSCs to injured cells has been reported. This finding indicates that mitochondria are important for self-renewal and differentiation of MSCs [33, 34]. Indeed, mitochondria have a characteristic ultrastructure and play a role in cell behavior. Some reports show that the differentiation potential of cells depends on the location of mitochondria [34, 35]. Furthermore, different cell types display different mitochondrial arrangements. For example, fibroblastic cells display more mature elongated mitochondria [36]. Folmes et al. [37] reported that reprogramming of cells to induced pluripotent stem cells results in mitochondrial rejuvenation. Moreover, mitochondria are exchanged between cells [38]. As shown in Fig. 6b, MSCs with mitochondria stained immediately before injection were transplanted into GFP-transgenic mice. After 2 days, the targeted organs with accumulation of MSCs were removed. In this experiment, adjacent continuous sections were considered to be the same section. One section was stained with an anti-GFP antibody and the adjacent section was stained with an antibody against MSC marker CD90. Host MSCs were thus distinguished from other cells. Transfer of mitochondria to endogenous MSC from transplanted MSCs was indicated by CD90(+) and GFP(−) MSCs in serial sections with red-stained areas of mitochondria. As a result, many CD90(+) and GFP(−) cells with stained mitochondria were located in the upper portion of the socket. Indeed, these results showed mitochondrial transfer from exogenous to endogenous MSCs in MRONJ model mice. These findings and previous reports suggest the importance of mitochondrial characteristics in cell differentiation and reprogramming. Mitochondrial characteristics appear to dictate cellular characteristics. However, the converse does not appear to be true. Therefore, it is possible that MSCs repair damaged cells by exchanging mitochondria [39]. Indeed, because a lot of organelles including a membrane protein complex were transferred with mitochondria, these contents should be also focused on the regulatory mechanism of erythropoiesis, biology, and clinical application of MSCs. However, one possibility is that mitochondria may participate in the treatment of host MSCs following administration of MSCs.

## Conclusions

Cell-based immunotherapy using systemic MSCs can potentially offer a safe and effective therapeutic modality in preventing the development of MRONJ disease. Furthermore, the mechanism of MSC treatment for MRONJ was shown to be the ability of transplanted MSCs to directly control and regulate d-MSCs in the patients. Moreover, mitochondria in the MSCs may play an important role in MSC regulation.

## Abbreviations

α-MSM, alpha-minimum essential medium; BP, nitrogen-containing bisphosphonate; BSA, bovine serum albumin; CFU-F, colony-forming unit-fibroblast; c-MSC, control mesenchymal stem cell; DAPI, 4′,6-diamidino-2-phenylindole; Dex, dexamethasone; d-MSC, diseased mesenchymal stem cell; EDTA, ethylenediaminetetraacetic acid; EdU, 5-Ethynyl-2′-deoxyuridine; FITC, fluorescein isothiocyanate; GFP, green fluorescent protein; GVHD, graft-versus-host disease; H & E, hematoxylin and eosin; i.v., intravenous; IL, interleukin; MRONJ, medication-related osteonecrosis of the jaw; MSC, mesenchymal stem cell; ONJ, osteonecrosis of the jaw; OVX, ovarectomy; PBS, phosphate-buffered saline; PFA, paraformaldehyde; SLE, systemic lupus erythematosus; TRITC, tetramethylrhodamine isothiocynate; Zol, zoledronate
